# Measuring Self-Reported Well-Being of Physicians Using the Well-Being Thermometer: Cohort Study

**DOI:** 10.2196/54158

**Published:** 2025-01-09

**Authors:** Marios Adamou, Sarah L Jones, Niki Kyriakidou, Andrew Mooney, Shriti Pattani, Matthew Roycroft

**Affiliations:** 1 University of Huddersfield, Huddersfield, United Kingdom; 2 South West Yorkshire Partnership NHS Foundation Trust, Belle Isle Health Park, Wakefield, WF1 5PN, United Kingdom, 44 1924 316492; 3 Leeds Beckett University, Leeds, United Kingdom; 4 Renal Unit, St James's Hospital, Leeds, United Kingdom; 5 London North West University Healthcare NHS Trust, London, United Kingdom; 6 Sheffield Teaching Hospitals NHS Foundation Trust, Sheffield, United Kingdom

**Keywords:** well-being, health care professionals, mental health, well-being thermometer, health care

## Abstract

**Background:**

Advancements in medical science have focused largely on patient care, often overlooking the well-being of health care professionals (HCPs). This oversight has consequences; not only are HCPs prone to mental and physical health challenges, but the quality of patient care may also endure as a result. Such concerns are also exacerbated by unprecedented crises like the COVID-19 pandemic. Compared to other sectors, HCPs report high incidence of stress, depression, and suicide, among other challenging factors that have a significant negative impact on their well-being.

**Objective:**

Given these substantial concerns, the development of a tool specifically designed to be used in clinical settings to measure the well-being of HCPs is essential.

**Methods:**

A United Kingdom–based cross-sectional pilot study was carried out to measure self-reported well-being in a cohort of 148 physicians, using the newly developed well-being thermometer. The aim of the tool is to allow respondents to develop an individual sense of “well-being intelligence” thus supporting HCPs to have better insight and control over their well-being and allow insights into how to manage it. The tool consists of 5 well-being domains—health, thoughts, emotions, spiritual, and social. Each domain can be measured individually or combined to produce an overall well-being score.

**Results:**

The tool demonstrated good internal consistency; the Cronbach α in this study was 0.84 for the total scale.

**Conclusions:**

Results from this cohort demonstrated that the well-being thermometer can be used to gather intelligence of staff well-being. This is a promising new tool that will assist HCPs to recognize their own well-being needs and allow health care organizations to facilitate change in policies and practices to reflect a better understanding of staff well-being.

## Introduction

Medicine has developed greatly in relation to disease control and health interventions, yet it is questioned if health care professionals (HCPs) are fully aware of their own well-being, and the toll poor levels of well-being may have on their lives [1]. HCPs face unique challenges that could be damaging to their mental and physical health [2-5], and given the recent challenges of the COVID-19 pandemic [6,7], plus the current public health crisis, monitoring the well-being of our HCPs is vital for the mental health of the workforce.

Poor well-being is often reported in HCP cohorts [8-11], leading to serious health consequences and reduced quality of life for those affected [12]. In economic terms, higher staff turnover and sickness absence are the consequence [13], potentially resulting in poor quality of care for patients [14]. HCPs consistently report higher levels of sickness absence, job dissatisfaction, and stress compared to other work sectors [15-20]. A recent systematic review found moderate to high levels of stress, anxiety, depression, sleep disturbance, and burnout reported by frontline health care employees compared to workers in other sectors [21]. Moreover, a survey conducted in the United Kingdom (pre–COVID-19 pandemic) reported that, of public sector workers, those who work for the NHS recorded the most stress [22]. There is also increasing morbidity in HCPs compared to the general population [23]. Reasons for these higher levels of stress are multifactorial, including lack of staff, increasing workload, patient expectation [23], emotional demands of the role [24], as well as others [25]. Research is supported by latest figures showing anxiety, stress, and depression are among the most reported reason for sickness absence in the NHS, accounting for 24.9% of absence in the year preceding September 2022 [26]. The issues pertaining to employee well-being have been acknowledged by the development of the “NHS workforce health and wellbeing framework” [27], although the outcomes of this are unclear at present. While there is extensive research around employee well-being [28], there are calls for deeper consideration of psychological needs of HCPs specifically [21,23,29]. The benefits of considering this not only help on an individual level, but also aid health care organizations with the economic cost of staff burnout [13].

Conceptualizing well-being is not straightforward. It is a complex construct, subject to much academic debate [30]. Current models of well-being tend to be grounded on concepts of mental illness or psychological functioning [31]. However, well-being is best described as multifaceted; measured by a range of subjective and objective concepts rather than one single notion [32]. Well-being is often used interchangeably with the term “mental health.” For instance, according to the World Health Organization (WHO), “mental health” is “a state of well-being in which every individual realizes his or her own potential, can cope with the normal stresses of life, can work productively and fruitfully, and is able to make a contribution to her or his community” [33]. It is important to note that well-being is different from the terms “quality of life” or “health-related quality of life,” terms that are primarily used to measure a person’s perspective of their own life within a cultural context [34]. Furthermore “happiness” is not conducive to well-being. Happiness is often tied to external factors such as pursuit and fulfillment of life goals or life events, rather than a holistic concept such as well-being [35,36]. In terms of how we define well-being here, there is no commonly accepted definition. Subsequently there is no universally accepted approach to measure it. Instead, studies of well-being are often ambiguous in their approach and theoretical underpinnings [37]. A systematic review of self-report measures for assessing well-being found that while the 2 main theories referred to were Diener’s model of subjective well-being and the WHO definition of health, authors were very rarely obvious about how theory had influenced the development of their tool or study. Further, argument suggests that the 2 most popular scales, The Warwick-Edinburgh Mental Wellbeing Scale and the WHO Five well-being index fail to capture the holistic nature of well-being [37].

Conceptualizing well-being is complex and often grounded in various subjective and objective factors. Despite various studies and tools aiming to measure well-being, none specifically target HCPs in a comprehensive manner. Adamou et al [31] developed a new theoretical framework of well-being specifically with HCPs in mind. The development of the well-being thermometer consisted of a three-step formation: (1) understanding the concept of well-being from existing literature and tools, (2) constructing a new framework of well-being, and (3) devising a tool to measure it. See Adamou et al [31] for further discussion of the conceptual development of the well-being thermometer. This study aims to pilot a new instrument, the well-being thermometer, specifically designed for HCPs. This tool aims to enhance individual “well-being intelligence,” helping HCPs better manage their mental and physical health.

## Methods

### The Well-Being Thermometer

Developed by Adamou et al [31], this tool incorporates 5 well-being domains—health, thoughts, emotions, spiritual, and social. It aims to provide a comprehensive yet individualized snapshot of well-being, thereby allowing for targeted interventions.

Each of the domains comprises 5 items related to that domain. Each domain can be reviewed individually or collectively providing the individual with a score, allowing reflection of overall and domain-specific well-being. The health domain includes items which relate to the physical and eating health. The thoughts domain relates to mental health. Emotions domain measures the experience of emotions such as joy, satisfaction, and frustration. Spiritual health aligns with the connection with oneself and the meaning of life. The social domain relates to the experience of positive relationships and social networks. Total score can range from 0 to 25, with a score of 25 being the highest level of well-being. Example statements include “I tend to dwell on things more than I should” or “I feel there is a lot to enjoy in life.” See Adamou et al [31] for further details.

### Participants and Procedure

Doctors were recruited to participate in the well-being survey through verbal advertisements at 2 separate events—a diploma course in occupational medicine organized by the Royal Society of Public Health and a Regional (Yorkshire) Conference for Physicians organized by the Royal College of Physicians. At each event, information was provided about the survey’s purpose and the automatic feedback participants would receive upon completion. The survey was administered using an online platform, and opportunity sampling was used to gather the cohort. Participants received an email containing a link to complete the survey online. By completing the survey, respondents were informed that they were consenting to have their data included in the research.

### Ethical Considerations

This project was granted ethics approval in line with the Research Ethics Policy and Procedures at Leeds Beckett University (138110). Consent for participation was implicitly granted by the subjects through their active engagement with the survey, after being fully informed about the study’s methodology and purpose via the online platform. Moreover, the study ensured the privacy and confidentiality of the participants by anonymizing or deidentifying all data used in the research. No compensation was provided to participants, aligning with the study’s observational and noninterventional design.

### Statistical Analysis

SPSS (version 29; IBM Corp) was used for statistical analyses. The Kolmogorov-Smirnov Test for Goodness of Fit determined data deviated significantly from normal distribution *P*<.05; therefore, nonparametric analysis was reported.

## Results

### Demographics

The cohort consisted of 148 physicians (without missing data), 68 (46%) recorded female sex, 78 (53%) reported male sex, and 2 (1%) respondents did not want to disclose gender. Age was recorded in category format, with age range of 40‐44 years recorded most frequently. Ages ranged from 20 to 69 years. Two respondents chose not to disclose age (see Table 1). Respondents recorded level and speciality pertaining to their profession. See Table 1 for full demographic details.

Table 1 shows the demographic information of the 148 participants who participated in this cross-sectional United Kingdom–based study exploring the validity of the well-being thermometer using survey data. Details include self-reported gender, age, profession level, and speciality pertaining to their medical career.

**Table 1.** Respondent demographics (N=148).

Demographic information		Values, n (%)
**Sex**		
	Female	68 (45.9)
	Male	78 (52.7)
	Prefer not to say	2 (1.4)
	Missing	0 (0)
**Age (years)**		
	20‐24	2 (1.4)
	25‐29	4 (2.7)
	30‐34	23 (15.5)
	35‐39	16 (10.8)
	40‐44	33 (22.3)
	45‐49	21 (14.2)
	50‐54	22 (14.9)
	55‐59	13 (8.8)
	60‐64	7 (4.7)
	65‐69	5 (3.4)
	Prefer not to say	2 (1.4)
	Missing	0 (0)
**Level**		
	Foundation doctor	3 (2)
	Core trainee	5 (3.4)
	Higher specialty trainee	20 (13.5)
	SAS^b^ or nontraining grade doctors	15 (10.1)
	Consultant or GP^a^	91 (61.5)
	Prefer not to say	5 (3.4)
	Other	9 (6.1)
	Missing	0 (0)
**Specialty**		
	Foundation programme	1 (7)
	Core medical training or internal medicine stage 1	3 (2)
	Acute internal medicine	12 (8.1)
	Cardiology	8 (5.4)
	Endocrinology and diabetes	4 (2.7)
	Gastroenterology	5 (3.4)
	General internal medicine	1 (0.7)
	Genitourinary medicine	11 (7.4)
	Geriatric medicine	33 (22.3)
	GP	22 (14.9)
	Infectious diseases	3 (2)
	Medical oncology	1 (0.7)
	Palliative medicine	5 (3.4)
	Rehabilitation medicine	1 (0.7)
	Renal medicine	2 (1.4)
	Respiratory medicine	14 (9.5)
	Rheumatology	3 (2)
	Sport and exercise medicine	1 (0.7)
	Stroke medicine	4 (2.7)
	Not applicable	3 (2)
	Prefer not to say	6 (4.1)
	Other	5 (3.4)
	Missing	0 (0)

a GP: general practitioner.

b SAS: specialty and specialist.

### Well-Being Scores

A total of 148 participants in this cross-sectional United Kingdom–based study recorded an overall median score of 18 (IQR 14-22) on the well-being thermometer.

Table 2 shows the cross-sectional median and IQR score recorded by the 148 participants of the United Kingdom–based study of the well-being thermometer. Scores for individual well-being domains ranged between 3 and 4.

**Table 2.** Scores by domain.

Domain	Median (IQR)	
Health	3 (2-4)	
Social	3.5 (3-5)	
Thoughts	4 (2-5)	
Emotions	4 (3-5)	
Spiritual	4 (3-5)	
All domains	18 (14-22)	

There was no significant effect of age, profession level, or speciality on well-being scores, both overall and domain specific.

### Gender-Based Analysis

For the United Kingdom–based cross-sectional study exploring the well-being thermometer, organized by gender (N=148), the median score for men (n=78) was 20 (IQR 16-23) compared to 16 (IQR 12-20.5) for women (n=68).

Table 3 shows the median and IQR scores recorded by the sample (N=148) on individual domains (health, social, thoughts, emotions, and spiritual) of the well-being thermometer, organized by gender. The data are derived from a cross-sectional United Kingdom–based study exploring the validity of the well-being thermometer. Scores ranged between 4 and 5 for men and 3 to 4 for women.

Men reported significantly higher levels of well-being than women overall (*U*=1593; *P*=.002). In terms of specific well-being domains, men reported higher levels of well-being on health (*U*=1979.5; *P*=.015), thoughts (*U*=1948.5; *P*=.019), emotions (*U*=1971; *P*=.013), and spiritual (*U*=1914; *P*=.014) domains, compared to women. There was also a trend for men to score higher on levels of social well-being (*U*=2109.5; *P*=.05).

**Table 3.** Domain scores by gender.

	Male (n=78)		Female (n=68)	
Domain	Median (IQR)		Median (IQR)	
Health	4 (3-5)		3 (1.5-4)	
Social	4 (3-5)		3 (3-4)	
Thoughts	4 (2-5)		3 (2-5)	
Emotions	4 (3-5)		3 (2-4)	
Spiritual	5 (4-5)		4 (3-5)	
All domains	20 (7)		16 (8.5)	

### Correlation

Table 4 demonstrates that Spearman rho identified positive relationships between scores on individual domains on the well-being thermometer in this cross-sectional United Kingdom–based study of HCPs (N=148). Suggesting that higher scores on 1 domain of well-being was reflected in other domains of well-being.

**Table 4.** Spearman rho.

		Total for health domain, n	Total for social domain, n	Total for thoughts domain, n	Total for emotions domain, n	Total for spiritual domain, n
**Total for health domain**						
	Correlation coefficient	—^a^	0.399^b^	0.376^b^	0.468^b^	0.400^b^
	Significance (2-tailed)	—	.00	.00	.00	.00
	n	—	144	143	144	142
**Total for social domain**						
	Correlation coefficient	0.399^b^	—	0.492^b^	0.453^b^	0.453^b^
	Significance (2-tailed)	.000	—	.000	.195	.000
	n	144	—	142	144	141
**Total for thoughts domain**						
	Correlation coefficient	0.376^b^	0.492^b^	—	0.640^b^	0.537^b^
	Significance (2-tailed)	.000	.000	—	.000	.000
	n	143	142	—	143	142
**Total for emotions domain**						
	Correlation coefficient	0.468^b^	0.453^b^	0.640^b^	—	0.534^b^
	Significance (2-tailed)	.000	.000	.000	—	.000
	n	144	144	143	—	143
**Total for spiritual domain**						
	Correlation coefficient	0.400^b^	0.453^b^	0.537^b^	0.534^b^	—
	Significance (2-tailed)	.000	.000	.000	.000	—
	n	142	141	142	143	—

a Not applicable.

b Correlation is significant at the .01 level (2-tailed).

### Cronbach α Analysis

Cronbach α values of 0.7 are considered high levels of internal consistency (DeVillis [38]; Kline [39]). Values above 0.5 are acceptable (Bowling [40]; Schmitt [41]). The scale had a high level of internal consistency, as determined by a Cronbach α of 0.872. Value would improve to α=0.878 if question 7 (I drink more alcohol than would be considered healthy) was removed.

## Discussion

### Principal Results

The aim of this study was to pilot a new instrument, the well-being thermometer. The tool was specifically designed for use with HCPs and was piloted here with a sample of physicians. In this cross-sectional study the well-being thermometer demonstrated a good level of internal consistency. It was evident that higher scores in 1 well-being domain correlated with higher scores on other well-being domains. The factors of age, speciality, and professional level had no significant effect on well-being. Results suggest that the well-being thermometer has the potential to be a useful and informative tool, both within clinical settings and on an individual level.

### Interpretations

The purpose of the well-being thermometer is to enhance individual “well-being intelligence.” To aid HCPs to better manage their mental and physical health, and to measure well-being in a more holistic framework than popular scales have allowed [31]. The well-being thermometer has shown to be a valid tool for measuring well-being in HCPs. Subsequently, with better understanding of well-being, services can offer better interventions, protection, and help to their workforce, and use this information to influence policy. The well-being thermometer also allows HCPs to identify elements of their individual well-being which may need attention.

Interestingly, results from this study demonstrated that there was a difference in well-being levels between the sexes, with men reporting higher levels of well-being than women overall, but also on the health, thoughts, emotions, and spiritual domains. This variable was considered important to explore here, as men have often reported higher levels of subjective well-being than women in numerous previous studies [21,29,42-44]. With best evidence derived from a large-scale study of 6397 HCPs, where men reported significantly greater level of overall well-being than women [45]. The findings from this study follow this trend.

Importantly, the well-being thermometer can be used to gather intelligence of staff well-being to facilitate change in policies and practices across health care organizations [31]. While better well-being is a valuable goal in its own right, HCPs should be a priority target because their roles require frontline responsibilities toward the general public and vulnerable populations. We have seen advancements in medical science largely focused on patient care, often overlooking the well-being of HCPs. This oversight has consequences—not only are HCPs prone to mental and physical health challenges, but the quality of patient care also endures as a result. Such concerns are exacerbated by unprecedented crises like the COVID-19 pandemic. HCPs are pressured with dealing with public health crisis in real time, in which difficult moral decision-making is associated with significant stress, lack of control, and feelings of fear [46]. Troublingly, the prevalence of burnout, depression, and suicide is high for this group [47-49]. Compromised well-being among HCPs leads to medical errors, reduced patient safety, high rates of staff turnover, increased absence due to sickness, and diminished patient care [12,14,50,51]. Thus, health care organizations have a responsibility to support and protect staff well-being for both patient and staff safety [52] and the well-being thermometer has shown it can be a useful tool in supporting this objective.

### Limitations

Further work pertaining to the well-being thermometer should be conducted with additional demographic information such as race and ethnicity, as this information was not captured here, and is a limitation of the study. A focus on threshold analysis is also necessary to gain further insight. Also, future work with larger samples is recommended.

### Conclusions

Conceptualizing well-being remains complex and often grounded in various subjective and objective factors, and despite various studies and tools aiming to measure well-being, none specifically target HCPs in a comprehensive manner. The aim of this study was to pilot a new instrument, the well-being thermometer, specifically designed for HCPs. This study demonstrated that the well-being thermometer can be used to gather intelligence of staff well-being. This information is required to facilitate much needed change in policies and practices across health care organizations. A specific focus on staff well-being will benefit not only HCPs, but also those who trust in our organizations to provide safe and efficient health care services.

## Abbreviations

HCP: health care professional
NHS: National Health Service
WHO: World Health Organization
